# Endometriosis of the Vermiform Appendix Presenting as a Tumor

**DOI:** 10.4021/gr2009.12.1330

**Published:** 2009-11-20

**Authors:** Tadashi Terada

**Affiliations:** Department of Pathology, Shizuoka City Shimizu Hospital, Miyakami 1231 Shimizu-Ku, Shizuoka 424-8636, Japan. E-mail: piyo0111jp@yahoo.co.jp

**Keywords:** Endometriosis, Appendix, Lymph nodes

## Abstract

Endometriosis of the vermiform appendix is a rare condition. Most patients with this disease are asymptomatic or present as acute or chronic appendicitis. The author herein reports a case of appendiceal endometriosis presenting as a tumor at the appendiceal oriffice. A 41-year-old woman complained of chronic abdominal pain. A colon endoscopy showed a tumor in the appendiceal orifice. Two biopsies of the tumor showed no remarkable changes. Imaging modalities including CT and MRI also revealed an appendiceal tumor. Resection of appendix, cecum, ascending colon, terminal ileum, and 16 lymph nodes were performed under the clinical diagnosis of gastrointestinal stromal tumor. Grossly, a tumor measuring 3 x 3 x 3 cm was recognized in the appendiceral orifice. Histologically, the tumor was endometriosis consisting of islands of endometrial glands and stroma. Immunohistochemically, the lesion was positive for estrogen receptor and progesterone receptor, but it was negative for p53 protein and Ki-67 labeling was very low (0.5%). Similar endometriosis-like glands or Mullerian duct remnants were recognized in six out of 16 regional lymph nodes. The present case suggests that appendiceal endometriosis may present as a tumor.

## Introduction

Endometriosis of the vermiform appendix is an uncommon condition [1-6]. Most patients with this disease are asymptomatic or present as acute or chronic appendicitis. Perforation of the appendix has been also reported [6]. However, appendiceal endometriosis presenting as a tumor is very rare. The author herein reports a case of appendiceal endometriosis presenting as a tumor.

## Case Report

**Figure 1:**
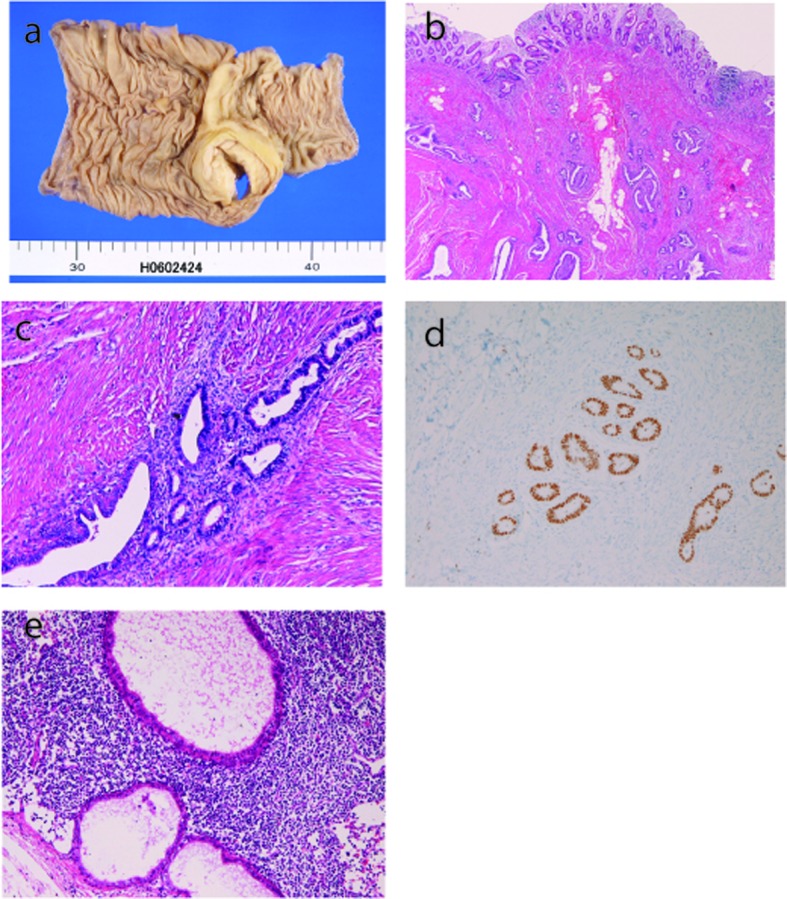
(a) Gross features. A tumor measuring 3 x 3 x 3 cm is recognized in the appendiceal orifice. (b) Low power view of the histology. Endometrial glands and stroma is scattered. HE, x 40. (c) High power view of the histology. The endometrial glands and stroma are evident. HE, x 200. (d) Estrogen receptor is expressed in the endometrial glands. Immunostaining, x 200. (e) Lymph node histology. Glandular structures of endometriosis or Mullerian duct remnants are recognized.

A 41-year-old woman complained of chronic abdominal pain. A colon endoscopy showed a tumor in the appendiceal orifice. Two biopsies of the tumor showed no remarkable changes. Imaging modalities including CT and MRI also revealed an appendiceal tumor. Resection of appendix, cecum, ascending colon, terminal ileum, and 16 lymph nodes were performed under the clinical diagnosis of gastrointestinal stromal tumor. Grossly, a tumor measuring 3 x 3 x 3 cm was recognized in the appendiceral orifice (Fig. 1a). Histologically, the tumor was endometriosis consisting of islands of endometrial glands and stroma (Fig. 1b, c). The endometriosis involved submucosa, proper muscular layer, and subserosa, sparing the mucosa. The muscular layer was very hypertrophic. Similar lesions without stroma were found in the six lymph nodes among the 16 lymph nodes dissected (Fig. 1d).

An immunohistochemical study was performed by Dako envision method (Dako Corp., Glostrup, Denmark) as previously described [7, 8]. The antigens examined were anti-estrogen receptor (ER) (clone 1D5, Dako), anti-progesterone receptor (PgR) (clone 1A6, Novocastra, Newcastle upon Tyne, UK), p53 protein (clone DO-7, Dako), and Ki-67 antigen (clone MIB-1, Dako). The endometrial tissue of the appendix was positive for ER (Figure 1E) and PgR, but negative for p53. The Ki-67 labeling was very low (0.5%). The lymph node lesions showed the similar immunohistochemical findings.

## Discussion

The present appendiceal tumor was relatively large. Histologically, it was composed of endometrial glands and stroma associated with muscular hypertrophy. Immunohistochecally, the tumor cells were positive for ER and PGR, and negative for p53 protein. The Ki-67 labeling was very low. Therefore, the present case is tumor-forming appendiceral endometriosis. The lymph nodes lesion also appears endometriosis, but may be Mullerian ducts remnants because no endometrial stroma was present.

Appendiceal endometriosis is a rare condition. Its frequency is less than 1% of patients with pelvic endometriosis [2]. Most patients present with acute appendicitis or asymptomatic [1-6]. The present case presented as an appendiceal tumor, and clinicians strongly suggested a gastrointestinal stromal tumor. Such a case appears very rare. The two biopsies failed to detect atypical glands. This is because the endometriosis did not involve appendiceal mucosa. Thus, appendiceral endometriosis should be taken into account in female patients when an appendiceal tumor is clinically found. Repeated biopsy may not be effective.

In summary, the author presented a case of appendiceal endometoriosis involving lymph nodes presenting an appendiceral tumor.
